# Extensile Exposures in Revision and Complex Primary Total Knee Arthroplasty: A Review of Anatomy, Biomechanics, and Techniques

**DOI:** 10.7759/cureus.50698

**Published:** 2023-12-17

**Authors:** Andrew Hadeed, Scott Sandilands, Teigen Goodeill, Max Jiganti, Jacqueline Krumrey, Nicholas S Tedesco

**Affiliations:** 1 Orthopedics, Good Samaritan Regional Medical Center, Corvallis, USA; 2 Orthopedic Surgery, Kendall Regional Medical Center, Miami, USA; 3 Orthopedic Surgery, Good Samaritan Regional Medical Center, Corvallis, USA; 4 Orthopedics and Traumatology, Good Samaritan Regional Medical Center, Corvallis, USA; 5 Orthopedic Oncology, Good Samaritan Regional Medical Center, Corvallis, USA

**Keywords:** extensile knee approach, surgical exposure, total knee, revision, arthroplasty

## Abstract

Developing adequate exposure when performing a revision total knee arthroplasty is critical to an efficient and safe intraoperative course. Proper planning and knowledge of the relevant anatomy are important when dissecting scar tissue associated with previous trauma or surgery and navigating bone loss. We present a review of the different total knee arthroplasty extensile exposure techniques that have been described in the literature. Specific exposures discussed include the femoral peel, banana peel, medial epicondylar osteotomy, quadriceps snip, tibial tubercle osteotomy, wandering resident, and the V-Y quadricepsplasty with patella turndown. Furthermore, we review the histological healing potential, biomechanical principles that drive post-operative expectations, post-operative rehabilitation protocols, and reported functional outcomes of each technique.

## Introduction and background

One of the many challenges in complex primary or revision total knee arthroplasty (rTKA), is establishing adequate surgical exposure to facilitate bone cuts, soft-tissue balance, appropriate patellofemoral mechanics, and accurate implant placement. One must consider previous skin incisions, preserving vascularity, degree of bone loss, joint stability, and pre-existing knee range of motion when planning a revision. Ways to potentiate healing include excising prior surgical scars, utilizing fasciocutaneous flaps, and avoiding narrow skin bridges between scars [1]. Over the years, multiple methods have been described to gain adequate exposure in rTKA. Ideally, the techniques listed below will not be necessary to successfully remove and insert total knee implants. In revision situations, there is an abundance of scar tissue, knee contractures, and distorted anatomy. It is paramount to exercise the basic principles of knee exposure before resorting to these extensile measures.

The authors' preferred technique for initial exposure of the knee includes a midline incision over the anterior knee followed by the creation of a full-thickness fasciocutaneous flaps 1-2 cm medial and lateral to the patella. One must follow the “web-like” loose connective tissue while elevating these flaps. At this point, a total anterior synovectomy is performed with the re-establishment of the medial and lateral gutters. A medial release of the proximal tibia is released up to the mid-portion of the medial tibial plateau with preservation of the MCL. Fibrous scar tissue is removed from the proximal lateral tibia and patellar tendon insertion. At this point, the patella can be subluxed laterally and the knee flexed. The surgeon should consider moving to one of the following extensile measures if implant removal is still restricted. The purpose of this article is to review the various exposure techniques used in rTKA to gain adequate exposure when a simple parapatellar arthrotomy is not sufficient, along with their post-operative rehabilitation protocols and reported long-term function.

## Review

Femoral peel

**Figure 1:**
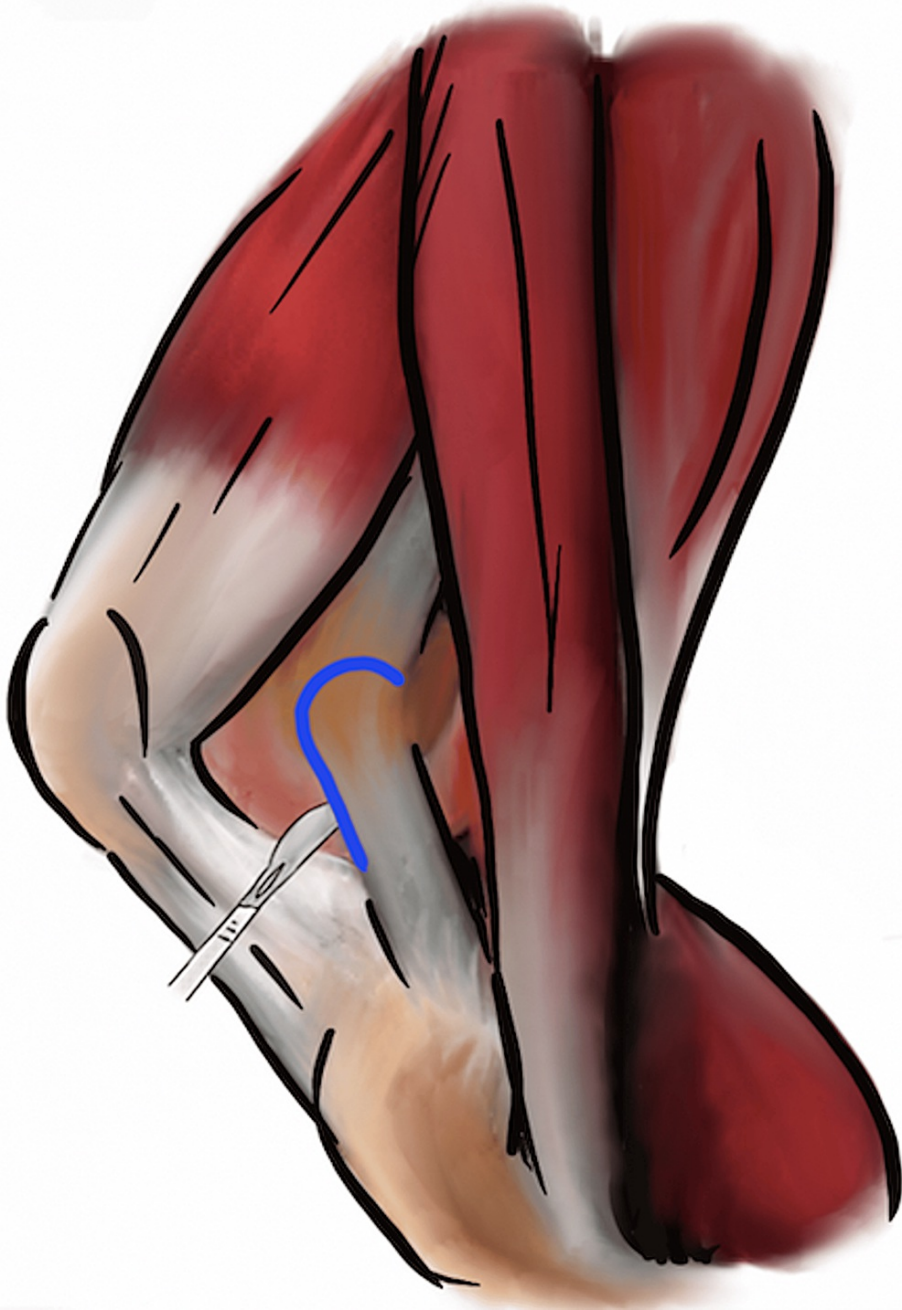
“Femoral peel” technique demonstrating a release of the medial collateral ligament off of the medial epicondyle of the femur. Image created by the authors.

The femoral peel technique was originally described in 1988 by Windsor and Insall for use in rTKA [2]. This technique is for exposing a stiff or ankylosed knee and involves a partial or complete subperiosteal dissection of the distal femur. The posterior capsule and adjacent scar tissue are initially elevated from the distal femur using electrocautery, and the subperiosteal dissection is carried proximally. With severe contractures, the medial and lateral gastrocnemius origin can be released along with the posterior capsule. This will leave the distal femur metaphysis and diaphysis void of all soft tissue and collateral ligament attachments (Figure 1) [3]. Maintaining a subperiosteal dissection posteriorly and keeping the knee in flexion will allow the popliteal neurovascular bundle to fall away from the posterior femur to help minimize this risk of injury. A varus-valgus constrained, total constrained prosthesis or a distal femoral replacement may need to be used in the case of detachment of the collateral ligaments depending on the degree of release.

In 2011, a modified version of the technique was described by Lavernia et al. [4]. Their technique starts by elevating the femoral insertion of the MCL with electrocautery in one flap with the medial capsule. Elevation of the posterior and lateral capsules can be performed if needed for more exposure or flexion contracture mitigation. The tibia can also be skeletonized in a similar manner by translating the tibia forward and subperiosteally elevating the posterior capsule off of the proximal tibia with electrocautery or periosteal elevator. The MCL and capsule can be elevated distally up to the pes anserinus. When using this technique, Lavernia et al. had a complication rate of 17% with the most common being post-operative deep infection, tibiofemoral dislocation, and periprosthetic fractures. Interestingly, 16 patients were reported to have a preoperative extensor lag of up to 45 degrees that resolved postoperatively. This was reported to be attributed to a tightening of the periarticular soft tissue sleeve surrounding the extensor mechanism [4].

In this series by Lavernia, patients were made weight-bearing as tolerated if the arthroplasty construct was stable. Physical therapy was initiated on postoperative day 1 and a continuous passive motion (CPM) device was used for the first three days. A knee immobilizer was used until the patient could perform a straight leg raise without assistance. The range of motion was limited to 90 degrees of flexion for the first eight weeks after surgery. The authors’ preferred rehabilitation plan is avoidance of the CPM by allowing passive extension and active flexion of the knee in the prone or standing position. A hinged knee immobilizer set at 0-90 degrees is utilized and locked in full extension during ambulation for the first six weeks. At six weeks, the patient is allowed the full range of motion, and active extension is begun. Strengthening is avoided until the full range of motion has been optimized, usually around the 8-10-week post-operative mark.

Medial epicondylar osteotomy

**Figure 2:**
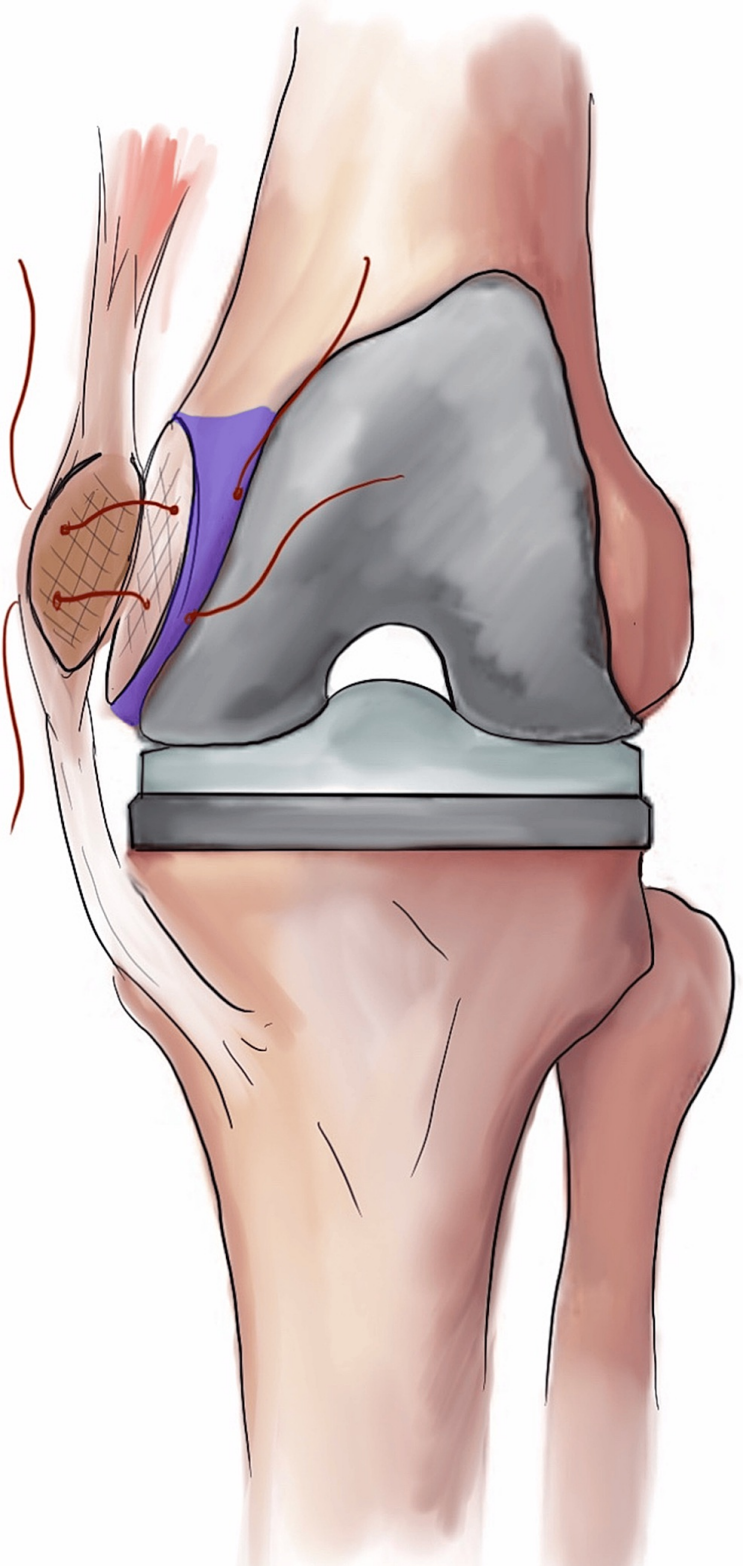
Technique for medial epicondylar osteotomy fixation. The area in purple demonstrates the bar of anterior femur that supports the osteotomy repair. Image created by the authors.

The medial epicondylar osteotomy is a technique initially described by Engh in 1999 and was utilized in primary and revision TKA and help correct severe varus deformities [5]. This technique starts with a midline incision and medially based arthrotomy. The knee is placed in 90 degrees of flexion and a segment that is 1 cm deep and 4 cm in diameter is osteotomized surrounding the medial epicondyle of the femur with a 1-¼ inch osteotome parallel to the long axis of the femur. This “wafer” of bone that is removed with the osteotomy contains the femoral attachment of the MCL and adductor magnus tendon. In a patient with less than 90 degrees of preoperative flexion, a quadriceps snip or tibial tubercle osteotomy (TTO) can be performed in conjunction. Since the distal and proximal soft tissue structures are still attached to the osteotomized wafer, the knee should be coronally stable in extension. The knee will not be stable in flexion until it is reattached to the distal femur. The osteotomized bone can be reattached in a slightly distal position on the medial epicondyle to help correct a varus alignment deformity if needed. The wafer is reattached with the knee at 90 degrees of flexion using #2 non-absorbable suture through the epicondylar fragment and secured to a thin bar of the anterior femur between the osteotomy and the distal femur cuts for the TKA (Figure 2). Cancellous screws can be placed if the wafer is still unstable after suture fixation. Postoperatively, range of motion and strengthening is encouraged. A knee brace is not necessary if the osteotomized medial epicondyle repair is stable. If the fragment cannot be secured, the knee should be placed in a knee brace for six weeks to allow healing of the epicondyle. Another option for persistent coronal instability would be to convert to a condylar constrained knee (CCK) prosthesis to allow immediate weight-bearing and range of motion without the use of a knee brace.

In the initial study by Engh, 60 patients (70 knees) underwent a medial epicondyle osteotomy and all patients achieved union at their two-year follow-up. Interestingly, 54% had a bony union whereas 46% had a fibrous union radiographically. There was no difference between the fibrous union and the bony union with respect to range of motion or pain. Heterotopic ossification developed at the union site in 25 of the 70 knees. There was no difference between the Knee Society Score or range of motion between the patients who developed heterotopic ossification and those who did not. There was no complication directly related to the osteotomy and none of the patients reported knee instability postoperatively [6].

Tibial tubercle osteotomy

**Figure 3:**
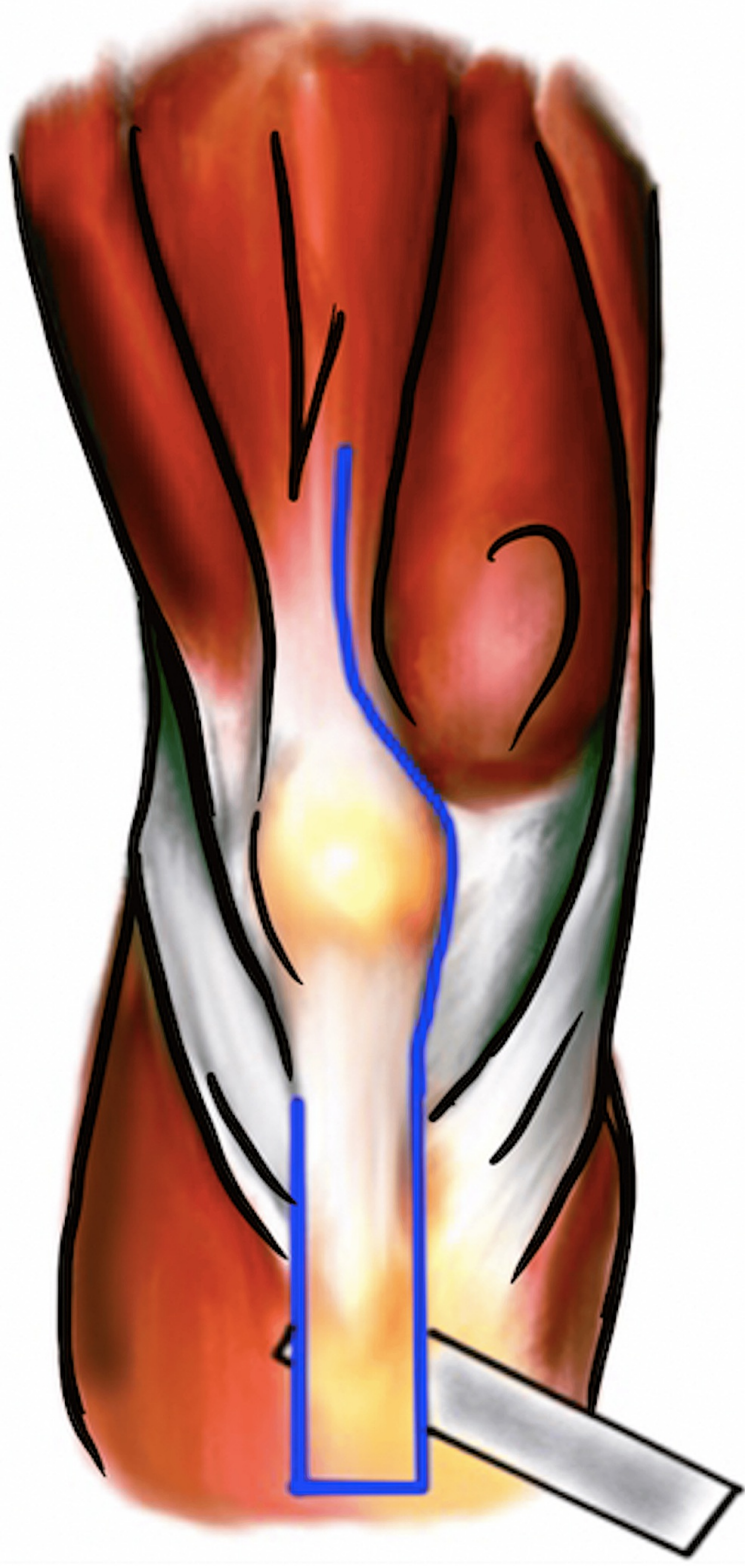
“Tibial tubercle osteotomy” technique performed with an osteotome. Image created by the authors.

The TTO was first described in 1983 by Dolin et al. [7]. It was further popularized by Whiteside et al. for performing revision TKAs to help with exposure of an arthrofibrosed knee or one with limited patellar mobility [8]. This technique starts with a standard midline incision that is extended eight to 10 cm distal to the tibial tubercle. After a medially based arthrotomy, an 8 cm long and at least 1.5 cm thick segment of anterior tibial crest and tibial tubercle is elevated using an oscillating saw or osteotome in a medial to lateral direction (Figure 3). The segment of osteotomized tibia remains attached to the lateral periosteum and anterior compartment musculature. The patella can be laterally subluxated and the knee flexed at this point to allow for visualization and removal of implants.

The best way to repair the osteotomy site is controversial. There are reports of suture or screw fixation, wire cerclage, plate fixation, and any combination thereof. Punwar et al. conducted a retrospective study of 42 knees who underwent TTO for rTKA. The osteotomy site was fixed with two diverging bicortical small fragment screws aimed around the tibial stem. Of their 42 knees, all osteotomies united and there were no reported extensor lags postoperatively. The range of motion increased from an average of 85 degrees preoperatively to 95 degrees postoperatively. However, two patients developed proximal migration of the osteotomy prior to union [9]. In a randomized prospective study by Bruni et al., the TTO and quadriceps snip exposures were compared in two-stage revision cases for prosthetic joint infections. The TTO was fixed with cerclage wiring. After an identical postoperative regimen, the TTO subgroup was found to have a statistically significant higher Knee Society Score and overall range of motion when compared to the quadriceps snip group [10].

**Figure 4:**
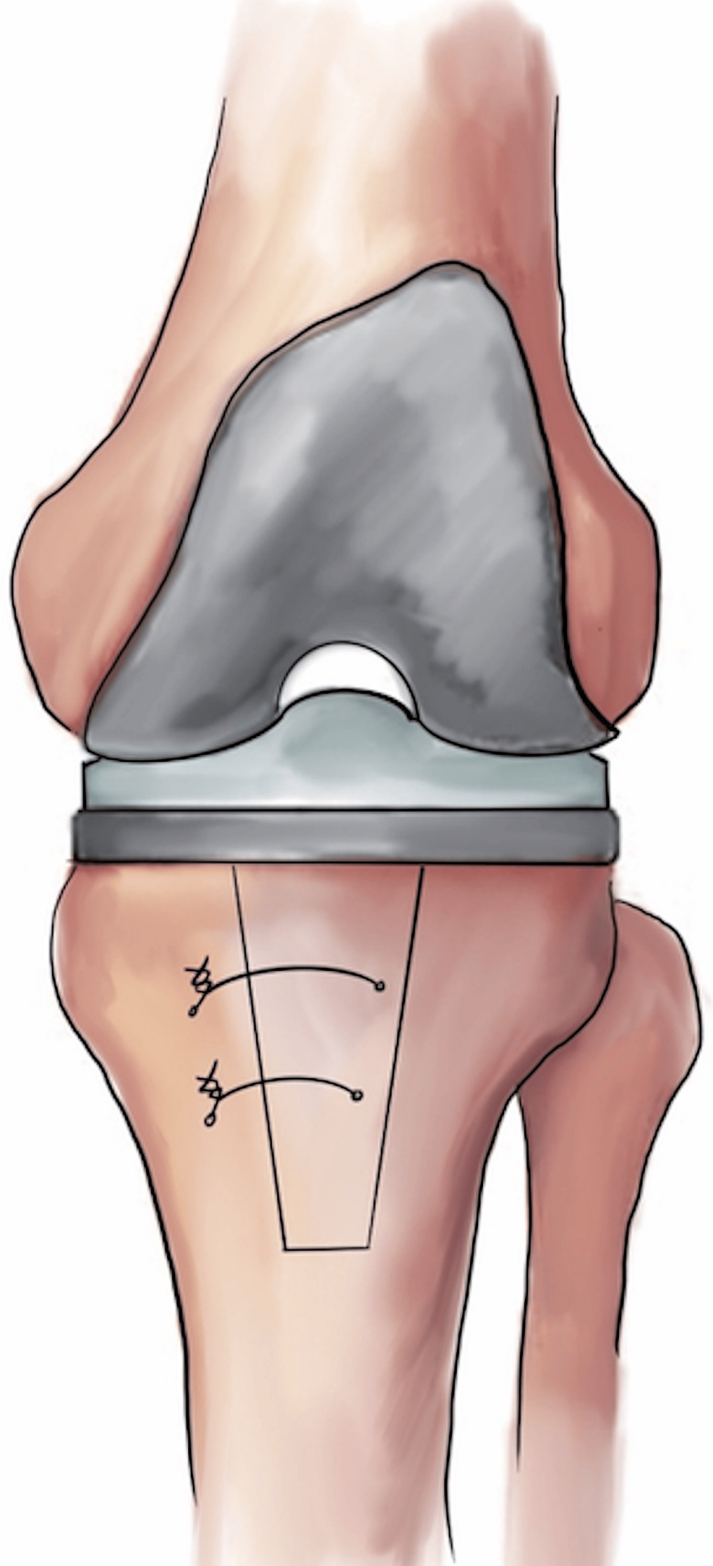
Fixation of the bony fragment with cerclage wiring at the completion of the tibial tubercle osteotomy. Image created by the authors.

The author’s preferred technique for repair of the osteotomized anterior cortex is with 18-gauge cobalt-chromium wires or #5 FiberTape™ (Arthrex, Naples, Florida). The fragment is first anatomically reduced while in the extended knee position. A 2.5-mm drill bit is placed just lateral to the midline on the osteotomized fragment aimed in a posteromedial direction. Two tunnels are made that connect the lateral fragment to the posteromedial proximal tibia and the wire or suture is tensioned over the anterior tibia (Figure 4). Postoperatively, the patient is placed in a knee brace locked in an extension for two weeks and allowed to bear weight as tolerated. At the start of week 2, the patient can begin active flexion from 0 to 20 degrees and with passive extension in the prone or standing position. Flexion can increase by 20 degrees each week subsequently. The knee brace should be locked in extension during ambulation until this time as well. At six weeks, the patient is allowed the full range of motion, and active extension is begun.

Quadriceps snip

**Figure 5:**
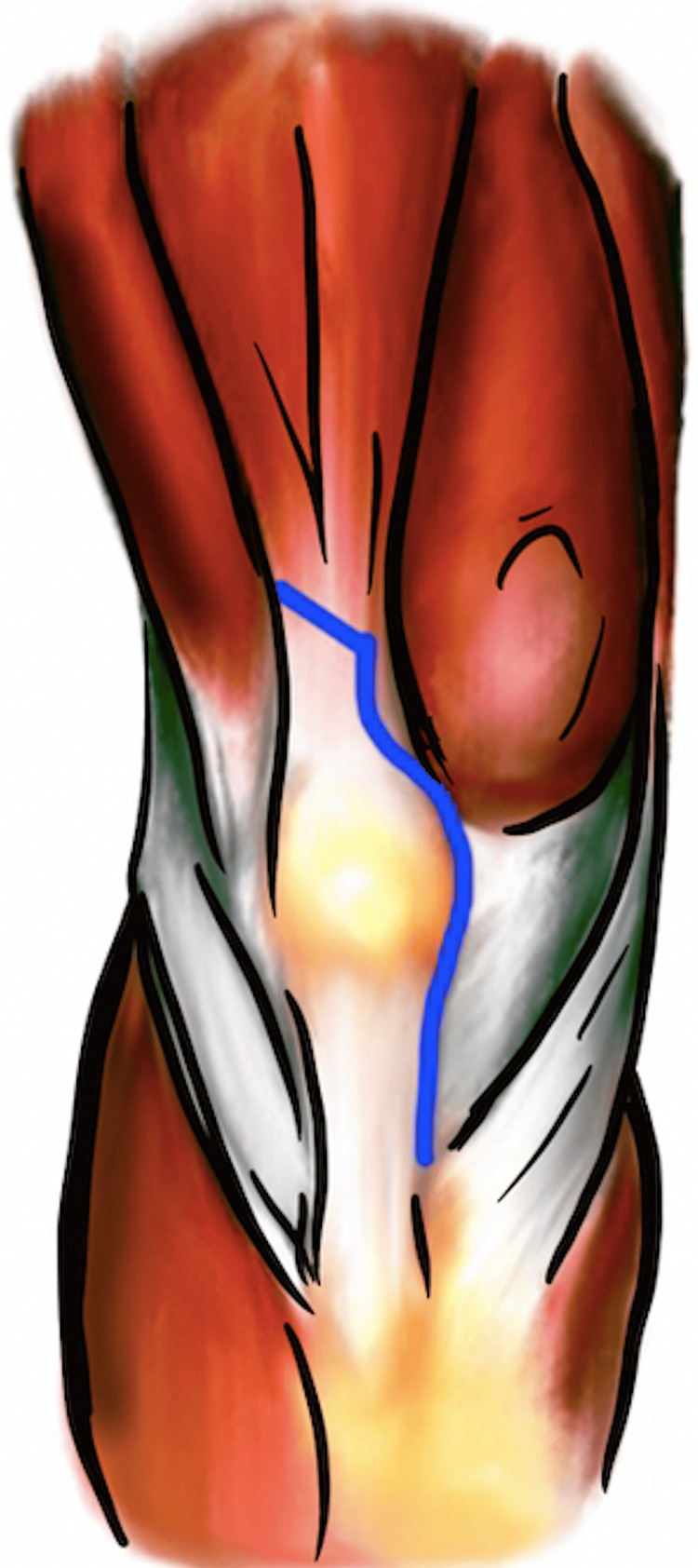
"Quadriceps snip" arthrotomy technique. Image created by the authors.

The quadriceps snip is a technique described in 1995 by Garvin and Insall for revision TKA in arthrofibrosed knees [11]. This technique is particularly useful for increasing exposure when the knee range of motion is less than 70 degrees of flexion. After a standard medial parapatellar arthrotomy, a 45-degree arthrotomy extension is made in a lateral and oblique fashion starting at the most proximal portion of the arthrotomy and into and in line with the fibers of the vastus lateralis (Figure 5). The lateral geniculate artery is preserved with this technique. If needed, a lateral retinacular release can be performed to allow full patellar eversion, if subluxation is restricted. After completion of the revision TKA, the oblique incision through the quadriceps tendon is repaired with a nonabsorbable suture in a figure-of-eight technique. The remaining arthrotomy is closed in a standard fashion.

The largest study to date was published by Abdel et al. in 2019, where 321 revision TKAs with a quadriceps snip were compared with a matched cohort who underwent a standard approach revision TKA. Postoperatively, both groups were made weight-bearing as tolerated if their bone reconstruction was stable and had an unrestricted range of motion. Resistive quadriceps exercise was delayed until eight weeks postoperative. They found no difference in the incidence of postoperative extensor lag, extensor rupture, KSS score, post-revision flexion contracture, implant survivorship, or overall complication rates [12]. A study by Bruni et al. in 2012, looked at 42 patients who underwent a rTKA with a quadriceps snip. Preoperatively, 28 patients did not have an extensor lag, seven patients had an extensor lag less than 15 degrees, and seven patients had an extensor lag greater than 15 degrees. Postoperatively, 23 patients had no extensor lag, 17 patients had an extensor lag less than 15, and two patients had a residual extensor lag greater than 15 degrees. They did not report any postoperative extensor mechanism disruptions among the 42 patients in this study who underwent quadriceps snips [10].

Banana peel

**Figure 6:**
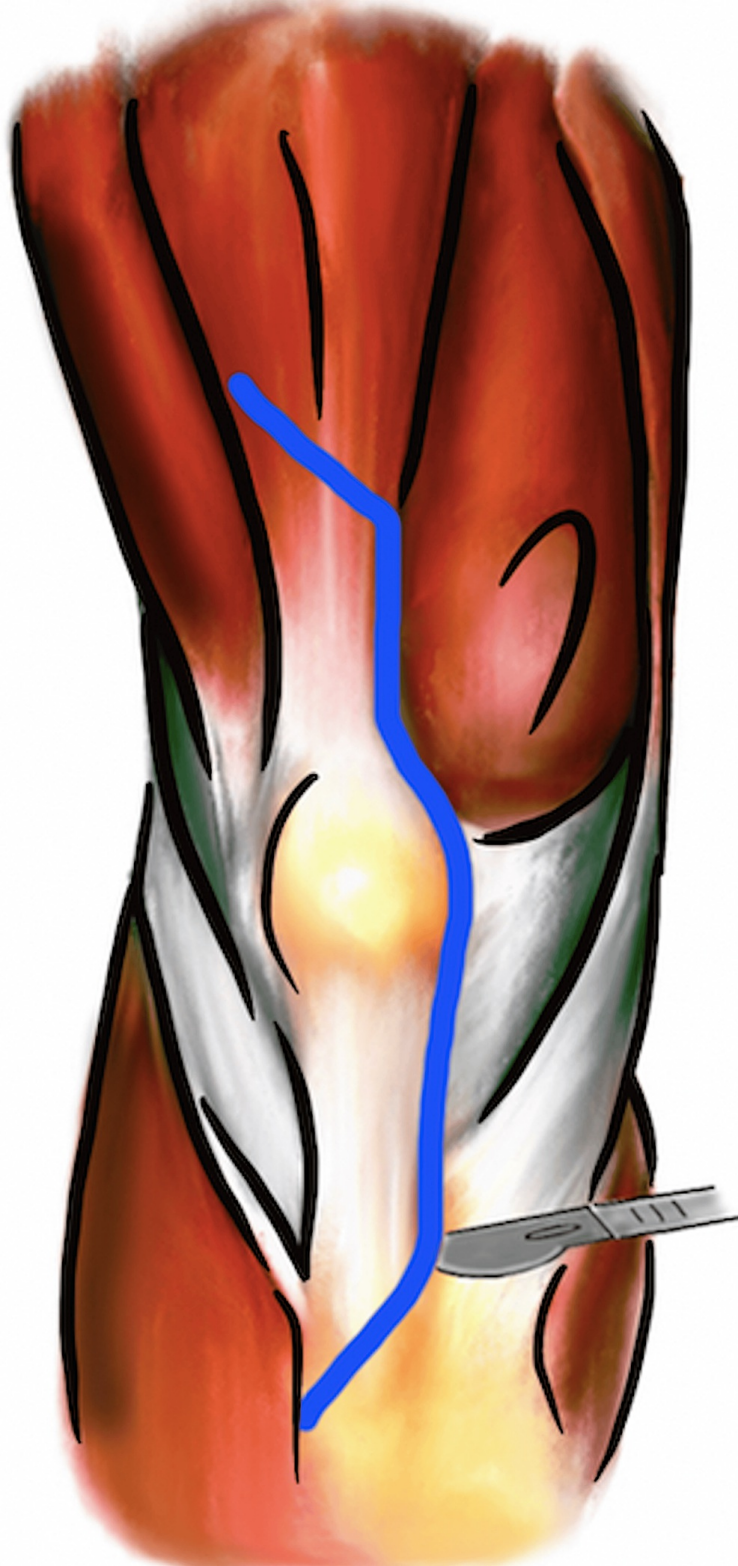
“Banana peel” technique demonstrating a proximal quadriceps snip and exposure of the proximal tibia by medial and distal release of the patellar tendon with preservation of the lateral soft tissue envelope and its patellar tendon attachments. The scalpel is not carried distal to the location in the figure. Instead, the patella is inverted, and the patellar tendon is peeled off its insertion the rest of the way. Image created by the authors.

The “banana peel” exposure technique in revision TKA was described in 2007 by Lahav and Hofmann. A standard medial parapatellar arthrotomy is made and dissection is carried along the medial patella tendon to the anterior tibia. A quadriceps snip is made through the quadriceps tendon as previously described. The patella is progressively inverted as a sleeve of tissue, capsule, and periosteum is elevated off the tibia along with the distal insertion of the patella tendon by tension alone (Figure 6). Neither sharp dissection nor electrocautery is needed to perform the patella tendon peel. The tendon can be reflected off of the tibial tubercle as much as needed but the most distal insertion and lateral border should be preserved. The extensor mechanism remains intact with this technique. A subperiosteal sleeve is reflected off the proximal tibia in a posteromedial direction to allow full exposure of the implants [13]. At the conclusion of the procedure, the tissue sleeves are approximated over the anterior tibia and closed using a nonabsorbable suture.

In the study by Lahav, 98 patients underwent an rTKA with the utilization of a banana peel exposure. The mean follow-up was 39 weeks, and no patients had an extensor mechanism disruption or extensor lag greater than 10 degrees. Complications reported in this study include arthrofibrosis requiring manipulation under anesthesia, aseptic loosening, and periprosthetic femoral fracture. The mean Knee Society Score was 176 and the mean postoperative arc of motion was 106 degrees. In a cadaveric study by Wall et al., the banana peel exposure and tibial tubercle osteotomy were compared based on load to failure and extensor mechanism integrity after cyclic loading. It was found that there was no difference in failure strength or patella tendon integrity after cyclic loading between the two exposures. This study provided evidence that both exposures can withstand the demand of postoperative weight-bearing [14].

The authors’ preferred technique is to restrict knee flexion greater than 90 degrees for six weeks via a hinged knee immobilizer to allow the patella tendon insertion to heal in situ before starting a more aggressive range of motion. However, further research is necessary to establish the ideal postoperative regimen.

Patellar turndown with or without V-Y quadricepsplasty

**Figure 7:**
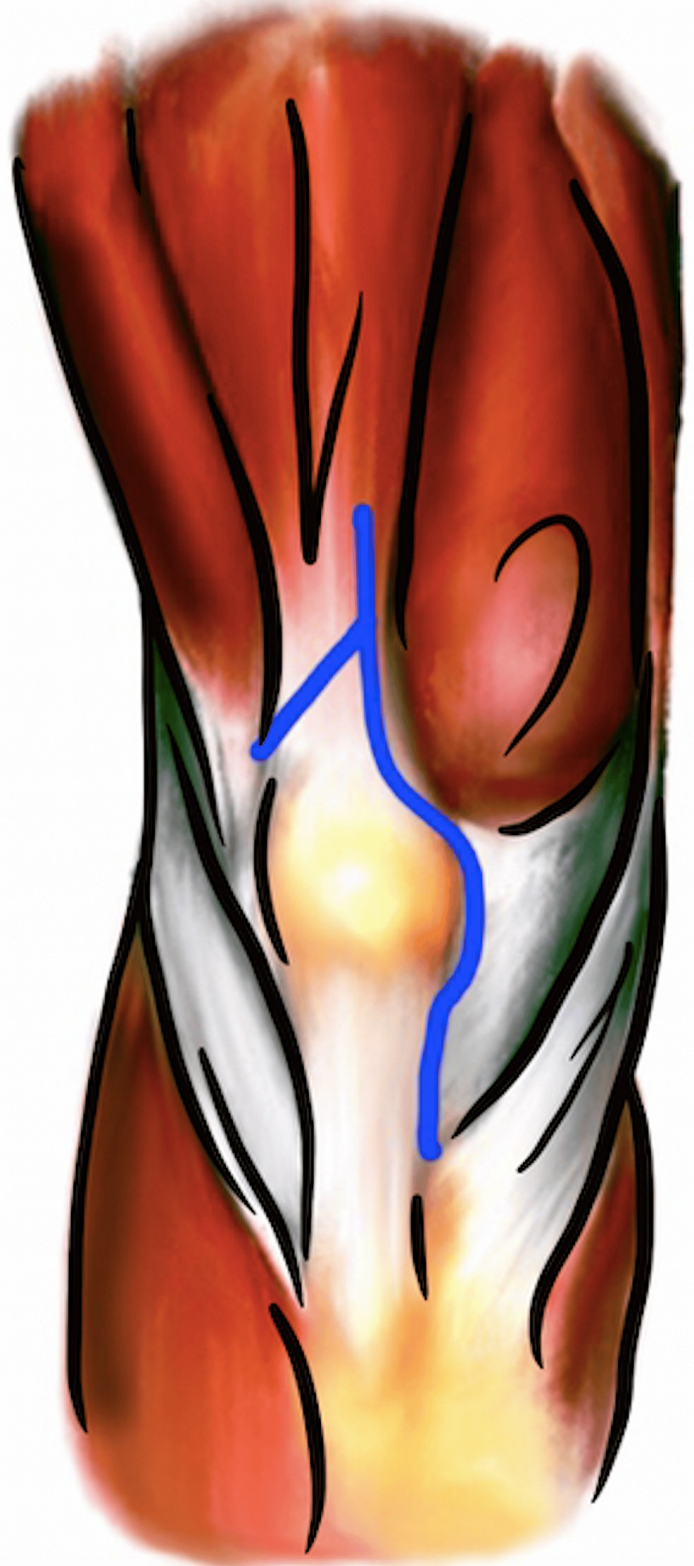
“V-Y quadricepsplasty with patella turndown” arthrotomy technique. Image created by the authors.

The V-Y quadricepsplasty with patellar turndown is a technique currently utilized in revision TKA and was first described in 1943 by Coonse and Adams. The indications for this procedure during this time are unclear but likely were used for the treatment of periarticular fractures of the knee. The original technique involved making an inverted V arthrotomy through the quadriceps tendon and reflecting the patella distally to gain exposure [15]. This V would then be reapproximated during closure or advanced in a V-Y fashion. A benefit of the V-Y Quadricepsplasty upon closure is the ability to lengthen the quadriceps tendon while preserving the patella tendon to proximal tibia continuity in cases of severe extension contracture or patella baja. In 1985, the technique was modified by Scott and Siliski, whereby they limited the lateral dissection along the insertion of the Vastus Lateralis musculature and preserved the inferior lateral genicular artery’s contributions to the patella [16]. In their technique, the medial limb of the inverted V arthrotomy occurs first with the initial parapatellar arthrotomy. The lateral limb is carried across the quadriceps tendon and down 3 cm along the border of the Vastus Lateralis in an inferolateral direction. The lateral limb can be carried down as far distally as the mid-pole of the patella (Figure 7).

At the completion of the procedure, a V-Y advancement closure can be made proximally with the length of the “Y” stem determined as needed on a case-by-case basis. The lateral limb can be left open to maintain lengthening or improve patellar tracking as needed. Our preferred closure involves flexing the knee to 90 degrees and repairing the Y limb and medial arthrotomy with number two nonabsorbable sutures. It is important to identify the maximum passive flexion of the knee intraoperatively after the capsular closure and to not exceed this degree in the first two to four weeks following surgery. For the first six weeks postoperatively, the patient is allowed to bear weight while in a brace locked in extension. For the first two weeks, they can come out of the brace daily for active flexion and passive extension in the prone or standing position, not to exceed the maximum flexion noted intraoperatively as above. Active extension and strengthening can be started as soon as two weeks post-operatively, as long as they have guided supervision. The extensor lag can be improved by keeping the brace locked in the extension at night [17]. At six weeks postoperatively, they are allowed to progress to full range of motion.

A study of 16 knees looked at the arm of motion after a rTKA with a V-Y quadricepsplasty. Motion increased from a mean of 9-77 degrees preoperatively to a mean of 4-85 degrees postoperatively. However, the extension strength of the operated knee was weaker compared to the normal contralateral knee by biomechanical testing [17]. Because of these suboptimal functional outcomes, this technique is best reserved for severely arthrofibrotic knees with limited range of motion.

Wandering resident approach

**Figure 8:**
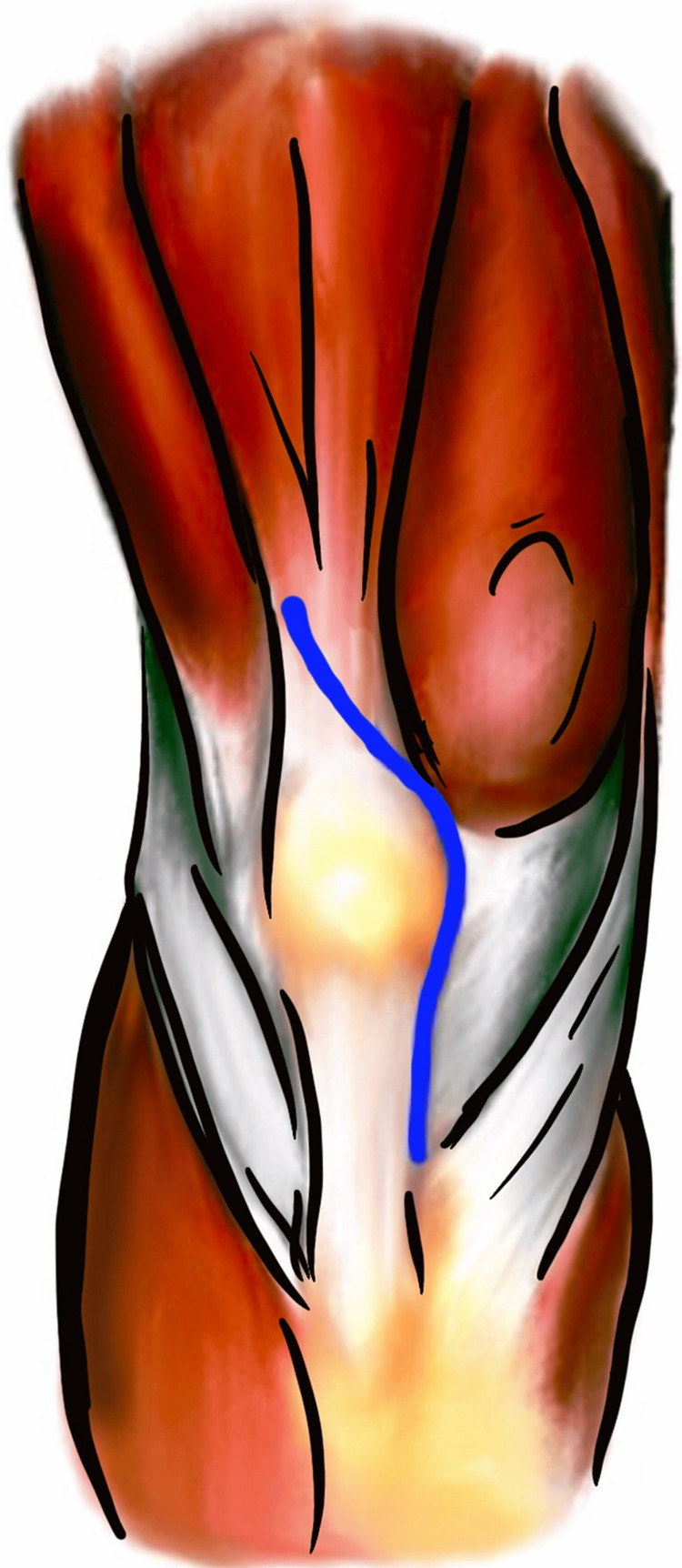
“Wandering resident” technique extensor mechanism incision. Image created by the authors.

The “wandering resident” surgical exposure is a technique that was described in 2004 by Hendel et al. and used for revision TKA in an ankylosed knee [18]. This exposure starts with a standard midline incision followed by a medial parapatellar arthrotomy. The arthrotomy continues distally along the medial border of the patella. Superiorly, the incision is carried obliquely in a superolateral direction across the quadriceps tendon (Figure 8). The arthrotomy and quadriceps tenotomy are followed by scar tissue release from the medial and lateral gutters until subluxation of the patella and flexion of the knee is achieved. Our preferred method of closure for the oblique quadriceps tenotomy is with #2 nonabsorbable, braided suture in a figure-of-eight fashion.

In the retrospective review by Hendel, 18 patients underwent revision TKA with a mean follow-up of three and a half years. In this study, the patients wore a knee immobilizer for six weeks with ambulation but were allowed to remove the brace daily for physical therapy and range of motion to 70 degrees of flexion. Full range of motion and weight bearing without the brace was started at six weeks. The mean postoperative range of motion and the Knee Society Score improved from 50 degrees and 40 preoperatively to 86 degrees and 84 postoperatively, respectively. One patient had a postoperative extensor lag of 10 degrees, but no other complications were encountered in this cohort related to the surgical technique [18].

## Conclusions

This article reviews several well-described techniques for additional exposure, their post-operative protocols, and their expected functional outcomes. All of the current methods have demonstrated acceptable complication rates and satisfactory results postoperatively. To fully understand the best type of exposure in revision TKA, further biomechanical, clinical, and histologic studies are needed to assess optimal healing, rehabilitation protocols, and functional outcomes.
